# Zinc chelator treatment in crush syndrome model mice attenuates ischemia–reperfusion-induced muscle injury due to suppressing of neutrophil infiltration

**DOI:** 10.1038/s41598-022-19903-0

**Published:** 2022-09-16

**Authors:** Yohei Haruta, Kazu Kobayakawa, Hirokazu Saiwai, Kazuhiro Hata, Tetsuya Tamaru, Hirotaka Iura, Gentaro Ono, Kazuki Kitade, Ken Kijima, Keiichiro Iida, Kenichi Kawaguchi, Yoshihiro Matsumoto, Kensuke Kubota, Takeshi Maeda, Dai-Jiro Konno, Seiji Okada, Yasuharu Nakashima

**Affiliations:** 1 Department of Orthopaedic Surgery, Graduate School of Medical Sciences, Kyushu University, 3-1-1 Maidashi, Higashi-ku, Fukuoka, 812-8582 Japan; 2 Department of Orthopaedic Surgery, Spinal Injuries Center, 550-4 Igisu, Iizuka, Fukuoka, 820-8508 Japan; 3 Department of Pathophysiology, Medical Institute of Bioregulation, Graduate School of Medical Sciences, Kyushu University, Fukuoka, 812-8582 Japan; 4 Department of Orthopaedic Surgery, Osaka University Graduate School of Medicine, 2-2 Yamada-oka, Suita, Osaka 565-0871 Japan

**Keywords:** Immunology, Molecular biology, Pathogenesis

## Abstract

In crush syndrome, massive muscle breakdown resulting from ischemia–reperfusion muscle injury can be a life-threatening condition that requires urgent treatment. Blood reperfusion into the ischemic muscle triggers an immediate inflammatory response, and neutrophils are the first to infiltrate and exacerbate the muscle damage. Since free zinc ion play a critical role in the immune system and the function of neutrophils is impaired by zinc depletion, we hypothesized that the administration of a zinc chelator would be effective for suppressing the inflammatory reaction at the site of ischemia–reperfusion injury and for improving of the pathology of crush syndrome. A crush syndrome model was created by using a rubber tourniquet to compress the bilateral hind limbs of mice at 8 weeks. A zinc chelator *N*,*N*,*N*′,*N*′-tetrakis-(2-pyridylmethyl)-ethylenediamine (TPEN) was administered immediately after reperfusion in order to assess the anti-inflammatory effect of the chelator for neutrophils. Histopathological evaluation showed significantly less muscle breakdown and fewer neutrophil infiltration in TPEN administration group compared with control group. In addition, the expression levels of inflammatory cytokine and chemokine such as IL-6, TNFα, CXCL1, CXCL2, CXCR2, CCL2 in ischemia–reperfusion injured muscle were significantly suppressed with TPEN treatment. Less dilatation of renal tubules in histological evaluation in renal tissue and significantly better survival rate were demonstrated in TPEN treatment for ischemia–reperfusion injury in crush syndrome. The findings of our study suggest that zinc chelators contributed to the resolution of exacerbation of the inflammatory response and attenuation of muscle breakdown in the acute phase after crush syndrome. In addition, our strategy of attenuation of the acute inflammatory reaction by zinc chelators may provide a promising therapeutic strategy not only for crush syndrome, but also for other diseases driven by inflammatory reactions.

## Introduction

Crush syndrome was first documented in Italy in 1908 during the Messina earthquake^1^. The mechanism of the disease was partially elucidated, based on histopathological findings, by Bywaters and Beall during World War II^2^. Seemingly non-critical conditions soon after rescue from compression due to collapsed debris could rapidly turn critical within a few hours^3^, so-called “smiling death” in the emergency field. This is often regarded as an important topic when a massive disaster occurs^4^.

The pathophysiology of crush syndrome is addressed as ischemia–reperfusion injury, which initiates the production of a large amount of reactive oxygen species (ROS) from vascular endothelial cells as a result of reoxygenation of the ischemic site. Subsequent muscle breakdown results in hypovolemic shock, acute renal failure with tubular necrosis, and lethal arrhythmias due to the release of toxic substances such as myoglobin and free potassium ions. The disease may progress to systemic inflammatory response syndrome, acute respiratory distress syndrome, and disseminated intravascular coagulation syndrome, which is characterized by systemic inflammation.

Inflammation is therefore deeply involved in the pathogenesis of ischemia–reperfusion injury in crush syndrome. Immediately after blood reperfusion into the ischemic muscle, the inflammatory response is initiated, and neutrophils are the first immune cells to infiltrate the affected muscles, which are activated within minutes^5^. While neutrophils are important as a first line of defense against infection in innate immunity, infiltrating neutrophils have been considered as one of the main factors in ischemic-reperfusion injury in various diseases, including myocardial infarction and cerebral infarction^6,7^. Infiltrating neutrophils release ROS and proteases, which harm the tissues and release inflammatory cytokines and chemokines to amplify the migration and activation of greater numbers of neutrophils into affected tissues, exacerbating the tissue damage^8,9^. Therefore, focusing on the reduction of neutrophil-mediated injury can be a good therapeutic strategy for ischemia–reperfusion injury in crush syndrome.

Zinc, a transition metal, is an essential trace element for an adequate and sufficient immune response to pathogens. In individuals deficient in zinc, granulocytes have been found to be significantly impaired in function^10^. Recently, it was shown that zinc signaling plays an essential role in multiple neutrophil functions, including activation, chemotaxis and extravasation^11,12^. In a study investigating the effect of in vitro chelation of Zn ions by *N*,*N*,*N*′,*N*′-tetrakis-(2-pyridylmethyl)-ethylenediamine (TPEN) on the activity of neutrophilic granulocytes, Hasan et al. demonstrated that TPEN-treated granulocytes impaired chemotaxis, granule release and cytokine production^13^.

In this study, we used an experimental animal model to investigate whether the suppression of inflammatory cell activity by the administration of a zinc chelator (TPEN) in the acute phase after reperfusion is effective for improving the pathophysiology of ischemia–reperfusion injury in crush syndrome.

## Results

### Vascular hyperpermeability, edematous changes, and neutrophil infiltration was observed in muscles with ischemia–reperfusion injury

**Figure 1:**
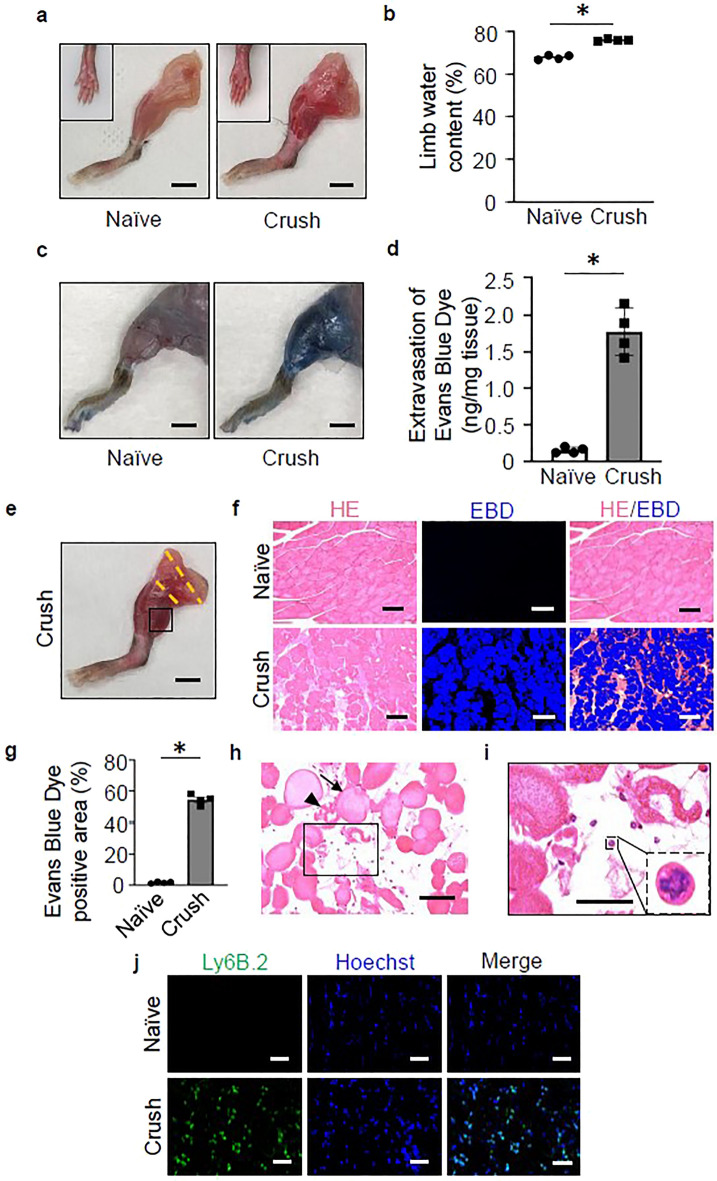
Vascular hyperpermeability, edematous changes, and neutrophil infiltration was observed in muscles with ischemia–reperfusion injury. (**a**) Representative images of the hind limbs after removal of the thigh and lower leg subcutaneous tissue with (crush group)/without (naïve group) tourniquet placement. Scale bars: 5 mm. (**b**) The quantitative analysis of the water content in the hind limbs of both groups at 3 h post-reperfusion (n = 4 per group). P < 0.005, unpaired *t* test. (**c**) Representative images of the hind limbs of naïve and crush model mice following the intraperitoneal injection of Evans Blue Dye (EBD). Scale bars: 5 mm. (**d**) The quantitative analysis of the extravasation of EBD in the hind limbs of both groups at 3 h post-reperfusion (n = 4 per group). P < 0.005, unpaired *t* test. (**e**) The muscle tissue was harvested from the gastrocnemius muscle, the region (the square part) distal to the compression site (between the orange broken lines) of the hind limb. Scale bar: 5 mm. (**f**) Representative images of the EBD uptake in the gastrocnemius muscle of the naïve and crush groups. Overlay image of the fluorescence image (EBD) and bright field image (Hematoxylin Eosin [HE]-stained). Scale bars: 100 μm. (**g**) The quantitative analysis of the EBD uptake in the gastrocnemius muscle of both groups at 3 h post-reperfusion (n = 4 per group). P < 0.001, unpaired *t* test. (**h**) Representative images of HE-stained muscle tissue sections in the crush group. Scale bar: 50 μm. The arrow indicates swelling of myofibers and the arrowhead indicates the breakdown of myofibers. (**i**) A representative expanded image of the inset of (**h**). A magnified view of the cell with nucleus between myofibers shown in the additional inset indicated with a broken line. Scale bar: 20 μm. (**j**) Representative images of muscle tissue specimens analyzed for neutrophils using Ly6B.2 (green) and Hoechst (blue) staining. Scale bars: 20 μm.

The affected limb in the Crush group at 3 h after reperfusion, following 2 h of compression, showed subfascial redness and swelling in comparison to the Naïve group (Fig. 1a). To determine the presence of swelling in the lower extremities on the body surface, the hindlimb plantar was observed (displayed in inset). The water content of the affected limbs in the Crush group was significantly increased in comparison to the Naïve group (Fig. 1b). We also evaluated the vascular permeability of the affected limbs by the intraperitoneal administration of Evans Blue Dye (EBD). The color of the affected limb changed to blue in the Crush group (Fig. 1c). The quantitative analysis showed significantly increased EBD-positive area in the Crush group (Fig. 1d). HE-stained sections of gastrocnemius muscle dissected from distal to the compression site (between orange dotted lines) in the Crush group (Fig. 1e, inset). Histological sections of lower leg muscles showed abundant leakage of EBD within the muscle fibers, even though these muscles were not compressed by the tourniquet (Fig. 1f). Quantitative analysis showed significantly increased EBD-positive area in the Crush group (Fig. 1g). HE-stained sections of gastrocnemius muscle in the Crush group showed more irregularity and edematous change in the myofibers (black arrow) and muscle breakdown (black arrowhead) (Fig. 1h). Lobular multinucleated cells were found in muscle tissue as shown in the inset with the broken line (Fig. 1i). Immunostaining of muscle tissue sections with an anti-neutrophil antibody (Ly6B.2) showed numerous Ly6B.2-positive neutrophils in the Crush group, whereas they were barely detectable in the Naïve group (Fig. 1j).

### Crush syndrome leads to renal injury and fluid therapy improves the survival rate

**Figure 2:**
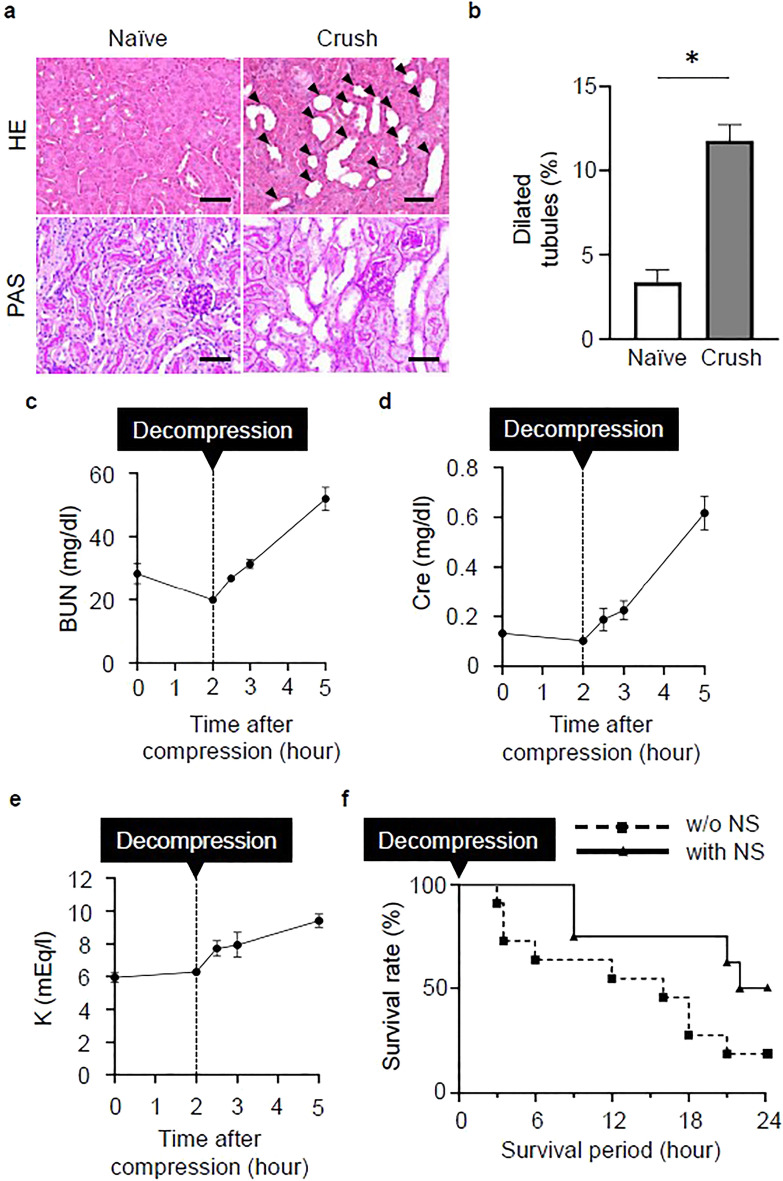
Crush syndrome leads to renal injury and fluid therapy improves the survival rate. (**a**) Representative images of HE and Periodic Acid Schiff (PAS)-stained renal tissue sections at 3 h post-reperfusion in the naïve and crush groups. Scale bars: 50 μm. (**b**) The quantitative analysis of the dilated tubules in the renal cortex in HE-stained sections from both groups (n = 4 per group). P < 0.05, unpaired *t* test. (**c**–**e**) Serum BUN, Cre, and K levels after reperfusion (n = 4 per group). *BUN* blood urea nitrogen, *Cre* creatinine, *K* potassium. (**f**) The post-reperfusion survival rates with/without normal saline (NS) treatment were calculated using the Log-rank test (Kaplan–Meier method). P < 0.05. *CS* crush syndrome, *NS* normal saline.

In the Crush group, HE- and PAS-stained kidney sections showed dilated renal tubules with thinning of the brush border (Fig. 2a). The proportion of dilated tubules in HE-stained sections was significantly higher in the Crush group than in the Naïve group (Fig. 2b). We examined the time course of parameters related to the renal function, and found that the blood urea nitrogen (BUN), creatinine (Cre), and potassium (K) levels were abnormally high (Fig. 2c–e). In crush syndrome, excessive vascular permeability results in a reduction of the circulating volume. Hypovolemia leads to decreased renal blood flow, which causes severe renal dysfunction. The survival rate of the Crush group was as low as 10% at 24 h (Fig. 2f). Many studies have reported that fluid infusion for compensating for the decrease in circulating volume is important^14^. Subcutaneous injection of 0.9% normal saline (NS) after decompression of the affected limb significantly improved the survival rate at 24 h (Fig. 2f).

### Zinc chelator treatment improved inflammation response and muscle breakdown in crush syndrome

**Figure 3:**
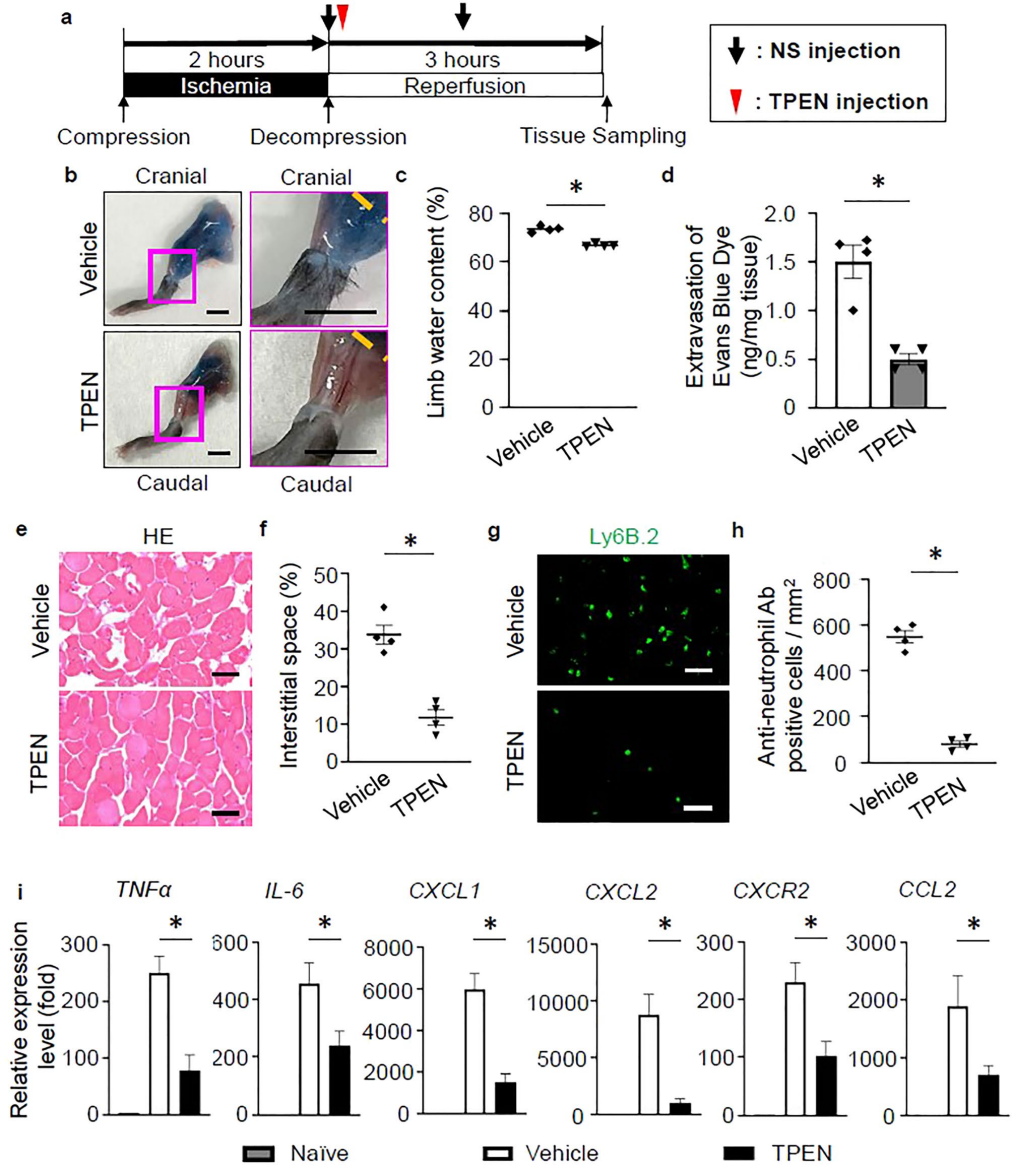
Zinc chelation therapy in crush syndrome reduces neutrophil infiltration, inflammation, and muscle breakdown. (**a**) The zinc chelator treatment protocol, *N*,*N*,*N*′,*N*′-Tetrakis (2-pyridylmethyl) ethylenediamine (TPEN) post-reperfusion. (**b**) Representative images of the lower legs with vehicle control and TPEN treatment, following the intraperitoneal injection of EBD. The lower legs with the fascia exposed at 3 h post-reperfusion. The orange dotted line indicates the distal end of the compression site of the hind limb. Scale bars: 5 mm. (**c**) The quantitative analysis of the water content in the hind limbs in the vehicle control and TPEN-treated groups at 3 h post-reperfusion (n = 4 per group). P < 0.05, unpaired *t* test. (**d**) The quantitative analysis of the extravasation of EBD in the hind limbs of both groups at 3 h post-reperfusion (n = 4 per group). P < 0.01, unpaired *t* test. (**e**) Representative images of HE-stained gastrocnemius muscle sections, the distal side of the compression site from mice in vehicle control and TPEN-treated groups. Scale bars: 50 μm. (**f**) The quantitative analysis of the interstitial space of the gastrocnemius muscle at mice in vehicle control and TPEN-treated groups (n = 4 per group). P < 0.05, unpaired *t* test. (**g**) Representative images of muscle tissue specimens stained with Ly6B.2 (green) in vehicle control and TPEN-treated groups (n = 4 per group). P < 0.005, unpaired *t* test. Scale bars: 50 μm. (**h**) The quantitative analysis of the Ly6B.2 (anti-neutrophil antibody)-positive cell count in vehicle control and TPEN-treated groups (n = 4 per group). P < 0.01, unpaired *t* test. (**i**) The TNFα, IL-6, CXCL1, CXCL2, CXCR2, CCL2 mRNA expression levels in vehicle control and TPEN-treated groups were compared (n = 4 per group). P < 0.05, unpaired *t* test.

Previous studies reported that neutrophil chemotaxis and activation can be inhibited by the zinc chelator, TPEN^12,13^. Therefore, we investigated whether TPEN treatment is effective for improving the pathology of crush syndrome through suppression of the inflammatory reaction. TPEN was dissolved in dimethyl sulfoxide (DMSO) and injected intraperitoneally 3 min after decompression of the limb. The solution containing TPEN (5 mg/kg of body weight) was diluted with DMSO and injected at a volume of 0.1 ml intraperitoneally into the mice (5 mg/kg of body weight/0.1 ml solution). The muscular tissues were dissected for evaluation at 3 h after reperfusion (Fig. 3a). First, we evaluated the vascular permeability of the affected limbs by the intraperitoneal administration of EBD. From the external appearance of the affected limb muscle at the lower thigh (the orange dotted line shows the distal border of the compressed area), EBD extravasation was observed in the vehicle control (Crush with NS + DMSO) group (Fig. 3b). In contrast, no EBD extravasation was observed in the TPEN-treated (Crush with NS + TPEN) group (Fig. 3b). The blue color change at the pressure area was the same for both groups (Fig. 3b). The water content of the affected limbs in the TPEN-treated group was significantly decreased in comparison to the vehicle control group (Fig. 3c). The quantitative analysis showed significantly increased extravasation of EBD in the TPEN-treated group (Fig. 3d). Histological sections of the lower leg muscles showed less muscle breakdown and interstitial space around myofibers in the TPEN-treated group (Fig. 3e). Quantitative evaluation of the interstitial space revealed that it was significantly decreased in the TPEN-treated group in comparison to the vehicle control group (Fig. 3f). Immunostaining of muscle tissue using an anti-neutrophil antibody (Ly6B.2) revealed numerous Ly6B.2-positive neutrophils in the vehicle control group, whereas few were detectable in the TPEN-treated group (Fig. 3g). Quantification of the Ly6B.2-positive cells around the myofibers revealed that the cell count was significantly decreased by TPEN-treated group in comparison to vehicle control group (Fig. 3h). The expression levels of inflammatory cytokines and chemokines (e.g., TNFα, IL-6, CXCL1, CXCL2, CXCR2, CCL2) in crush syndrome were significantly suppressed in TPEN-treated group (Fig. 3i).

### Zinc chelator treatment improved the renal function and survival rate in crush syndrome

**Figure 4:**
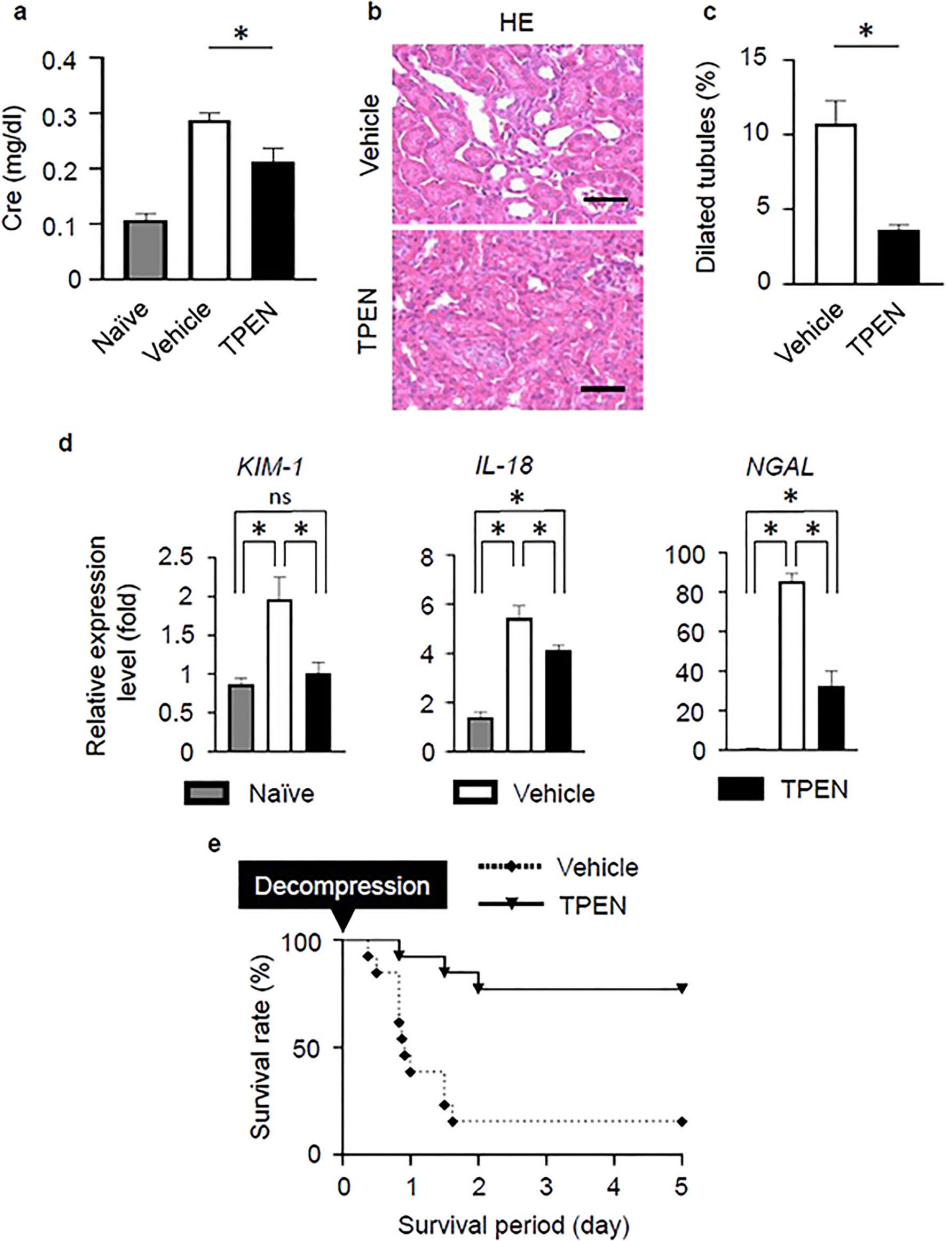
Zinc chelation therapy improves the renal function and survival rate in crush syndrome. (**a**) The effects of TPEN treatment on the serum Cre levels at 3 h after reperfusion (n = 4 per group). P < 0.05, unpaired *t* test. (**b**) Representative images of HE-stained renal tissue in vehicle control and TPEN-treated groups at 3 h post-reperfusion. Scale bars: 50 μm. (**c**) The quantitative analysis of the dilated tubules in the renal cortex in HE-stained sections in vehicle control and TPEN-treated groups (n = 4 per group). P < 0.05, unpaired *t* test. (**d**) The KIM-1, IL-18, NGAL mRNA expression levels in 3 groups (Naïve, vehicle control, TPEN-treated) were compared (n = 4 per group). P < 0.05, one-way ANOVA with the Tukey–Kramer post hoc test. (**e**) The post-reperfusion survival rate in vehicle control and TPEN-treated groups (n = 13 per group). P < 0.001, Log-rank test.

The serum Cre level at 3 h after reperfusion was found to be markedly suppressed in TPEN-treated group (Fig. 4a, P < 0.05). Renal tissues in the TPEN-treated group showed less dilatation of the tubules on HE-stained sections in comparison to the vehicle control group (Fig. 4b). There was a significant reduction of tubular dilatation in the TPEN-treated group (Fig. 4c). The mRNA expression levels of early markers of kidney damage (KIM-1, IL-18, NGAL) in the renal tissue of the crush syndrome model were significantly suppressed in the TPEN-treated group (Fig. 4d). The survival rate of the TPEN-treated group was > 90% at 5 days after reperfusion; thus, the prognosis was obviously improved in comparison to the vehicle control group (Fig. 4e).

## Discussion

In the present study, we revealed that the administration of a zinc chelator immediately after reperfusion suppressed neutrophil infiltration, the expression of inflammatory cytokines, and muscle breakdown, resulting in the improvement of the renal function and the survival prognosis in a mouse model of lower-extremity muscle crush syndrome.

Besides trauma, crush syndrome is the most common cause of death after an earthquake^15^, and based on lessons learned at emergency medical facilities in earthquake-prone countries and regions^16^, these have been recognized as “preventable deaths”. The pathogenesis of crush syndrome includes hypovolemic shock, renal failure, electrolyte abnormalities, lethal arrhythmias, and systemic inflammatory response syndrome due to rhabdomyolysis. In order to improve these pathological conditions, patients receive intravenous fluid therapy and hemodialysis^16^. However, some patients still develop renal failure, which may lead to death^17,18^.

In previous reports, acute kidney injury (AKI) has been reported to occur in as many as 41.6% of crush syndrome victims^19^; these patients require hemodialysis and have a mortality rate of approximately 20%^20^. The main causes of renal damage in crush syndrome are renal hypoperfusion due to hypovolemia and nephrotoxic substances such as myoglobin and ROS^21^. However, the only currently available treatment options are massive infusion of fluids for hypovolemia and hemodialysis for nephrotoxic substances^22^.

The cause of renal hypoperfusion is considered to be a reduction in the effective circulating volume due to excessive fluid transfer from the intravascular space to the interstitial space^23,24^. In the present study, extravascular leakage of EBD was observed in the muscle tissue not only at the site of compression but also on the distal side of the compressed area of the affected limb (Fig. 1c), which indicates increased vascular permeability caused by ischemia–reperfusion injury. Since the water content of the affected hindlimbs was also higher than that of sham operated hindlimbs (Fig. 1b), increased vascular permeability leads to the development of water retention in the affected limbs, leading to hypovolemia and renal hypoperfusion. The pathogenesis of this increased vascular permeability is the activation of vascular endothelial cells by the production of ROS^25^ from ischemic muscle tissue after reperfusion. Moreover, inflammatory cells in the peripheral blood infiltrate the injured area, amplifying the inflammatory response and further increasing vascular permeability.

As for nephrotoxic substances, myoglobin is a well-recognized nephrotoxic substance contained within muscle cells that is associated with the development of acute renal failure^26^. In crush syndrome, the rapid reperfusion of blood to ischemic muscle tissue due to compression leads to the production of ROS and the subsequent amplification of the inflammatory response, resulting in muscle damage^27^. Although regulation of reperfusion itself is difficult, the inflammatory response induced by reperfusion is controllable, and suppression of the inflammatory response is expected to reduce muscle damage and suppress the leakage of myoglobin into circulation. In this study, we confirmed that EBD permeated into the myofiber of muscle with ischemia–reperfusion injury. Since EBD is a membrane-impermeable dye that binds to serum albumin, EBD-positive myofiber indicates the disruption of its membrane, which causes the release of intracellular substances such as myoglobin into circulation. Therefore, focusing on the suppression of the inflammatory reaction to attenuate hyperpermeability and subsequent muscle damage could lead to novel treatment options for ischemia–reperfusion injury.

As for the inflammatory response induced by ischemia–reperfusion injury, neutrophils are the first cells to infiltrate the injury site^28,29^. Infiltrating neutrophils in the damaged muscle tissue are activated, releasing myeloperoxidase, proteases (e.g., elastase), and ROS, thereby amplifying the muscle damage^30–32^. Therefore, we hypothesized that with the direct inhibition of neutrophil infiltration and activation, it may be possible to terminate the uncontrolled amplification of the inflammatory response and subsequent muscle breakdown, and improve the pathogenesis of crush syndrome.

The essential element zinc plays a critical role in the immune system, acting as an element of cellular signal transduction. Therefore, the function of neutrophils is impaired by decreased zinc levels^11,12,33^. The intracellularly permeable zinc chelator TPEN has been reported to inhibit neutrophil migration and activation by depleting intracellular free zinc ions^12,13^. In this study, the administration of TPEN immediately after reperfusion suppressed neutrophil infiltration and the expression of inflammatory cytokine/chemokine in the muscle tissue of affected hindlimbs (Fig. 3g–i). The administration of TPEN also attenuated the leakage of EBD in ischemia–reperfusion injured muscle (Fig. 3b), indicating reduced vascular permeability and less disruption of the myofibers.

As for previously proposed treatments for crush syndrome, carbon monoxide-enriched red blood cells have been shown to be effective against myoglobin and heme protein^34^. Allopurinol^35^ and hydrogen sulfide^36^ have been shown to be effective for blocking the pathways producing oxidative stress. Dexamethasone^37^, anti-HMGB-1 antibodies^17,38^, lactoferrin^39^, icing^40^, and ulinastatin^41^ have shown efficacy against the inflammatory cascade. In this study, TPEN was also shown to have anti-inflammatory effects in ischemia–reperfusion muscle injury. Since TPEN is an intracellularly permeable agent that acts within seconds on the involved immune cells^42^, the systemic administration of TPEN immediately after reperfusion can efficiently attenuate the amplification of the inflammatory response, which would lead to the prevention of the systemic inflammatory response.

Our study demonstrated, for the first time, that the administration of TPEN in the acute phase attenuates ischemia–reperfusion muscle injury in crush syndrome by inhibiting neutrophil activation and migration. This may be a potential therapeutic strategy for improving the clinical outcomes not only in cases of ischemia–reperfusion injury in crush syndrome but also in the field of transplantation medicine (e.g., liver, kidney, and heart transplantation), post-traumatic vascular reconstruction, and musculocutaneous flap surgery, which may cause shock and acute renal failure in the perioperative period^43^.

## Conclusions

The findings of our study suggest that zinc chelators could contribute to the resolution of myoglobin-induced acute renal injury after crush syndrome by suppressing neutrophil infiltration in ischemia–reperfusion injured muscle. In addition to crush syndrome, the use of zinc chelators to attenuate the acute inflammatory reaction may be a promising therapeutic strategy for other diseases induced by ischemia–reperfusion injury.

## Methods

### Animal model of crush syndrome

Adult C57BL/6J wild-type mice (8 weeks old, 18–21 g) were used in this study. The mice were kept under a constant 12 h light/dark cycle at a constant room temperature (23 ± 2 °C) with ad libitum access to food and water.

All animal studies were approved by the Committee of Ethics on Animal Experimentation of our institute and conducted in accordance with ARRIVE guidelines (https://arriveguidelines.org). Every effort was made to reduce the number of animals used and to minimize their suffering.

**Figure 5:**
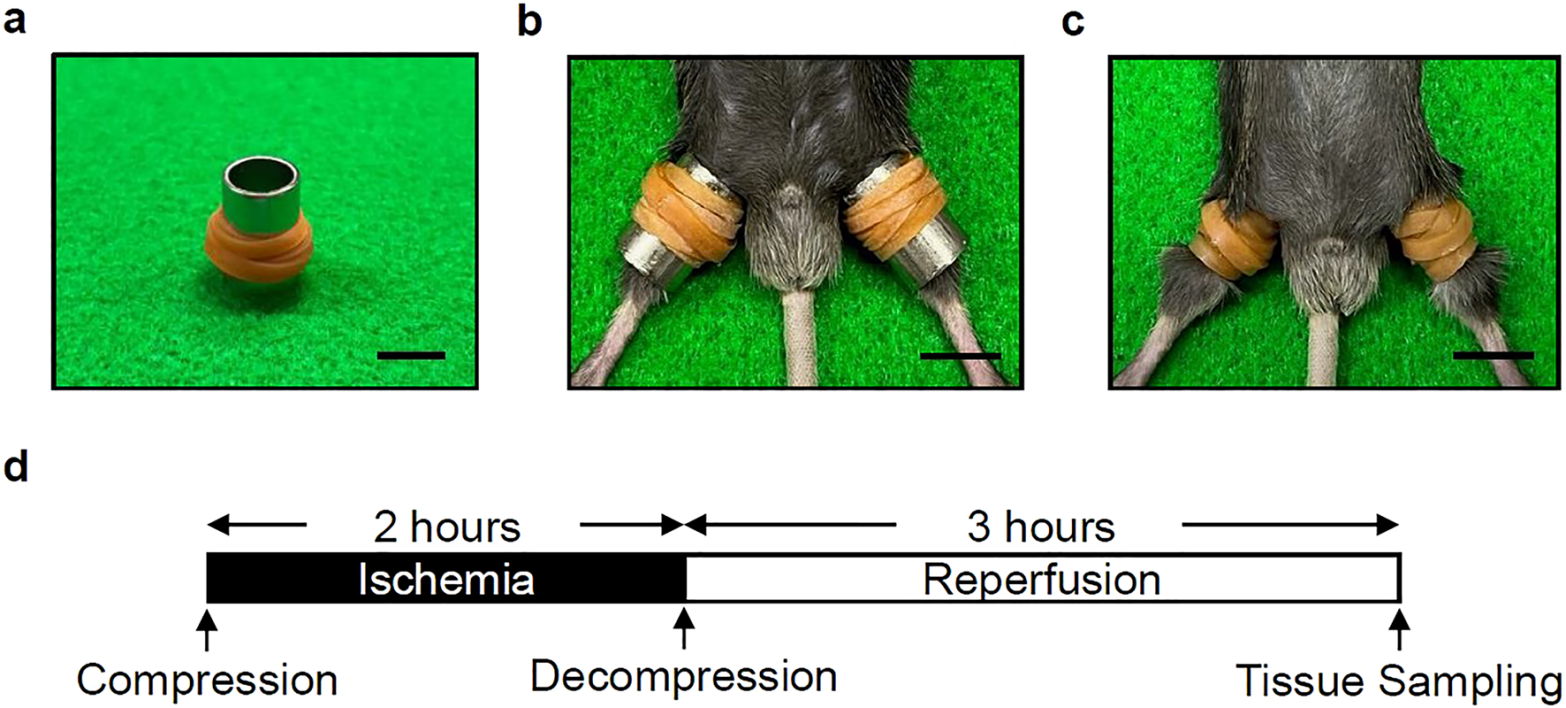
Establishment of the mouse model of crush syndrome and the experimental protocol. (**a**) Equipment for the insertion of the rubber tourniquet. Scale bar: 1 mm. (**b**) Insertion of the tourniquets by sliding on the pipe. Scale bar: 1 mm. (**c**) Representative positioning of the rubber tourniquets on both hind limbs. Scale bar: 1 mm. (**d**) Ischemia of the hind limbs caused by the tourniquet persists for 2 h. The hind limbs are collected at 3 h post-reperfusion (after release of the tourniquet).

The mice were anesthetized with the intraperitoneal administration of pentobarbital (75 mg/kg) to make the procedure painless, and were then placed in the supine position. A natural rubber tourniquet of 3 mm in width and 1 mm in thickness was applied around a stainless steel pipe of 10 mm in diameter, and the end of the band was glued in accordance with a previously reported method^14^ (Fig. 5a). Both hind limbs were placed on compression devices (Fig. 5b), and the stainless pipe was pulled out, leaving the rubber tourniquet (Fig. 5c), to create crush injuries in both hind limbs. The compression tension was defined as the stress at which the 11-cm rubber tourniquet was stretched and wrapped 6 times around a pipe. After 2 h of compression, the rubber tourniquet was cut off with fine scissors to produce a reproducible ischemia reperfusion phenomenon, and samples were obtained at 3 h after reperfusion in accordance with the protocol (Fig. 5d). After reperfusion, the body temperature and hemodynamics were controlled, even after awakening from anesthesia, and it was confirmed that the animals could move without pain. Animals that developed significant autophagia were excluded from the study.

### Zinc chelator administration

*N*,*N*,*N*′,*N*′-Tetrakis (2-pyridylmethyl) ethylenediamine (TPEN) was dissolved in dimethyl sulfoxide (DMSO) and injected intraperitoneally 3 min after decompression of the limb. The solution containing TPEN (5 mg/kg of body weight) was diluted with DMSO and injected at a volume of 0.1 ml intraperitoneally into the mice (5 mg/kg of body weight/0.1 ml solution). The TPEN solution, once diluted with DMSO, was not additionally diluted at all until it was injected into the mouse.

### Limb water content test

Limb edema was evaluated by analyzing the water content of the limb. Injured hindlimbs were dissected on the cranial side of the compressed area (distal from proximal end of the femur), weighed, and then dried for 72 h at 80 °C to determine the dry weight. The percentage of water content in limb tissue was calculated using the following formula according to the method of Saiwai et al.^44^:

$$ {\text{hindlimb}}\;{\text{water}}\;{\text{content}}\;(\%) = ({{\text{wet}}\;{\text{weight }} - {\text{ dry}}\;{\text{weight}}})/{\text{wet}}\;{\text{weight }} \times 100\;(\%). $$

### Evaluation of the renal function and electrolyte abnormalities

Blood samples were taken from the transorbital venous plexus at 0, 2, 2.5, 3, and 5 h after compression of both hind limbs. After blood collection, the samples were allowed to stand at room temperature for 30 min, centrifuged (room temperature, 1500×*g*, 10 min), and the serum components were transferred to another tube and stored at − 80 °C. The serum urea nitrogen (BUN), creatinine (Cre), and potassium (K) levels were measured over time.

### Evans Blue Dye administration and permeability assay

Mice were intraperitoneally injected with 80 µg/g B.W. Evans Blue Dye (EBD; Sigma-Aldrich) in phosphate-buffered saline (PBS). At 3 h after injection, the mice were anesthetized and perfused with PBS for 5 min. After perfusion, the injured hindlimbs were dissected at gastrocnemius muscle (distal to the compressed area) and homogenized (15,000 rpm, 60 s) in 0.5 ml of 50% trichloroacetic acid. The sample homogenate was diluted 1:1 in 100% ethanol and centrifuged at 15,300×*g* for 10 min. The supernatant was collected and absorbance (620 nm) was measured using an EnSight plate reader.

At 3 h after reperfusion, the animals were re-anesthetized and transcardially fixed with 4% paraformaldehyde, and thereafter the gastrocnemius muscle and kidney were removed, and embedded in paraffin. Sections (thickness: 3 μm) were cut. Muscle and renal tissue specimens were subjected to hematoxylin–eosin (HE) staining, while renal tissue was subjected to periodic acid Schiff (PAS) staining. All images were obtained using a BZ-9000 Digital Microscope System (Keyence, Osaka, Japan). The degree of renal dysfunction was determined by measuring the area of tubular dilatation in five arbitrarily selected locations in a 400 × field of view in kidney sections using the Image J software program.

The muscle tissue was also embedded in an optimal cutting temperature compound following put in 10% sucrose in PBS for 24 h and 30% sucrose in PBS for 24 h. The embedded tissue was immediately frozen in liquid nitrogen and stored at − 20 °C until use. Frozen sections of the muscle were cut on a cryostat in the axial plane at 14 µm, mounted on glass slides. Muscle sections were permeabilized with 0.01% Triton X-100 and 10% normal goat serum in PBS, pH 7.4, for 60 min. The primary antibody, rat anti-neutrophil (Ly6B.2, clone 7/4, 1:400, Serotec, Oxford, UK), was applied to sections at 4 °C overnight. The sections were then incubated with the secondary antibody, Alexa Fluor 488-conjugated goat anti-rat IgG (1:1000, Invitrogen, Carlsbad, CA). Negative control experiments included a normal muscle section incubated with the primary and secondary antibody, and an injured muscle section incubated with the secondary antibody alone or with non-specific rat IgG and the secondary antibody. Fluorescence-labeled sections were coverslipped with Fluorescent Mounting Medium (S3023; Dako Cytomation, Glostrup, Denmark).

### Evans Blue Dye uptake of the tissue

After the injection of EBD, paraffin embedded muscle tissue sections were prepared and stained with HE. Images of HE-stained sections obtained in brightfield were overlaid with the autofluorescence of EBD observed under a fluorescence microscope (620/680 nm).

### Survival of the mouse model of crush syndrome

We collected the time points at which mice died after decompression.

### Quantitative real-time reverse transcription PCR

We isolated total RNA from the injured gastrocnemius muscle and kidney using an RNeasy Kit (Qiagen, Hilden, Germany). RNA was primed with oligo dT primer and reverse transcribed using PrimeScript reverse transcriptase (TaKaRa, Shiga, Japan). Real-time reverse transcription (RT)-PCR was performed using primers specific to the genes of interest (Table 1) and TB Green Premix Ex TaqII (TaKaRa) in 20 µl of reaction solution. The mRNA levels were normalized to the glyceraldehyde-3-phosphate dehydrogenase (GAPDH) mRNA level for each sample. cDNA was synthesized from mRNA extracted from these tissues. RT-PCR was conducted using a Thermocycler (Biometra, Gottingen, Germany) and the products were detected by electrophoresis and ethidium bromide staining.

**Table 1:** Primers used for quantitative RT-PCR.

Gene symbol	Accession number	5′-Forward primer-3′	5′-Reverse primer-3′
IL-6	NM_031168.2	GCTCTCCTAACAGATAAGCTGGAG	CCACAGTGAGGAATGTCCACAAAC
TNFα	NM_013693.3	TTATGGCTCAGGGTCCAACTCTGT	TGGACATTCGAGGCTCCAGTGAAT
CXCL1	NM_008176.3	GGGAGGCTGTGTTTGTATGTCTTG	CGAGACCAGGAGAAACAGGGTTAA
CXCL2	NM_009140.2	CGGATGGCTTTCATGGAAGGAGTG	GCTAAGCAAGGCACTGTGCCTTAC
CXCR2	NM_009909.3	TCTGGCATGCCCTCTATTCTG	AAGGTAACCTCCTTCACGTAT
CCL2	NM_011333.3	CCTGGATCGGAACCAAATGAGATC	CCGAGTCACACTAGTTCACTGTCA
KIM-1	NM_134248.2	AACATCTACACACACATGGACT	TGCTTCAGTGTGATTACTCCAG
IL-18	NM_008360.2	ACTGTACAACCGCAGTAATACGG	AGTGAACATTACAGATTTATCCC
NGAL	NM_008491.1	CCCTGTATGGAAGAACCAAGGA	CGGTGGGGACAGAGAAGATG
GAPDH	NM_008084.2	GACTTCAACAGCAACTCCCACTCT	GGTTTCTTACTCCTTGGAGGCCAT

*RT-PCR* reverse transcription polymerase chain reaction.

### Statistical analysis

Results were expressed as the mean ± standard error of the mean (SEM) at least three independent experiments. Data were compared between two groups using an unpaired *t* test. A one-way ANOVA with the Tukey–Kramer post hoc test was applied for multiple comparisons of the RT-PCR results obtained with renal tissue. Survival curves were calculated by the Kaplan–Meier method with the level of significance set at P < 0.05, and survival was compared by a log-rank test. In all statistical analyses, *P* values of < 0.05 were considered to indicate statistical significance. All statistical analyses were performed using the Graph Pad Prism software program (version 9.2.0, GraphPad Software, San Diego, CA, USA).

### Ethics approval

Ethical approval was obtained from the ethical review committee of Kyushu University Graduate School of Medical Sciences (A21-466-0), in accordance with the provisions of the institution’s Regulation for Animal Experiments.

## Data Availability

The datasets generated and/or analyzed in the current study are available upon request from the corresponding author.

## Abbreviations

CS: Crush syndrome
EBD: Evans Blue Dye
HE: Hematoxylin eosin
PAS: Peroidic acid-Schiff
BUN: Blood urea nitrogen
Cre: Creatinine
K: Potassium
NS: Normal saline
TPEN: *N*,*N*,*N*′,*N*′-Tetrakis (2-pyridylmethyl) ethylenediamine
DMSO: Dimethyl sulfoxide
PBS: Phosphate-buffered saline
SEM: Standard error of the mean
GAPDH: Glyceraldehyde-3-phosphate dehydrogenase
TNFα: Tumor necrosis factor α
IL-6: Interleukin-6
CXCL1: C-X-C motif chemokine 1
CXCL2: C-X-C motif chemokine 2
CXCR2: C-X-C chemokine receptor type 2
CCL2: Chemokine (C-C motif) ligand 2
KIM-1: Kidney injury molecule 1
IL-18: Interleukin-18
NGAL: Neutrophil gelatinase-associated lipocalin

## References

[1] von Colmers F (1909). Uber die durch das Erbeben in Messina. Arch. fur klin. Chir..

[2] Bywaters EGL, Beall D, Bywaters GL, Knochel P (1941). Crush injuries with impairment by of renal. J. Am. Soc. Nephrol..

[3] Murata I (2011). Characterization of systemic and histologic injury after crush syndrome and intervals of reperfusion in a small animal model. J. Trauma Inj. Infect. Crit. Care.

[4] Vanholder R (2007). Earthquakes and crush syndrome casualties: Lessons learned from the Kashmir disaster. Kidney Int..

[5] Schofield ZV, Woodruff TM, Halai R, Wu MCL, Cooper MA (2013). Neutrophils—A key component of ischemia–reperfusion injury. Shock.

[6] Baxter GF (2002). The neutrophil as a mediator of myocardial ischemia–reperfusion injury: Time to move on. Basic Res. Cardiol..

[7] Frieler RA (2017). Genetic neutrophil deficiency ameliorates cerebral ischemia–reperfusion injury. Exp. Neurol..

[8] Chen LW, Chang WJ, Chen PH, Liu WC, Hsu CM (2008). TLR ligand decreases mesenteric ischemia and reperfusion injury-induced gut damage through TNF-α signaling. Shock.

[9] Metzemaekers M, Gouwy M, Proost P (2020). Neutrophil chemoattractant receptors in health and disease: Double-edged swords. Cell. Mol. Immunol..

[10] Keen CL, Gershwin ME (1990). Zinc deficiency and immune function. Annu. Rev. Nutr..

[11] Hujanen ES, Seppä ST, Virtanen K (1995). Polymorphonuclear leukocyte chemotaxis induced by zinc, copper and nickel in vitro. BBA Gen. Subj..

[12] Hasan R, Rink L, Haase H (2013). Zinc signals in neutrophil granulocytes are required for the formation of neutrophil extracellular traps. Innate Immun..

[13] Hasan R, Rink L, Haase H (2016). Chelation of free Zn2+ impairs chemotaxis, phagocytosis, oxidative burst, degranulation, and cytokine production by neutrophil granulocytes. Biol. Trace Elem. Res..

[14] Rosenthal SM (1943). Experimental chemotherapy of burns and shock. Public Health Rep..

[15] Ukai T (1997). The great Hanshin-Awaji earthquake and the problems with emergency medical care. Ren. Fail..

[16] Atef-Zafarmand A, Fadem S (2003). Disaster nephrology: Medical perspective. Adv. Ren. Replace. Ther..

[17] Shimazaki J (2012). Systemic involvement of high-mobility group box 1 protein and therapeutic effect of anti-high-mobility group box 1 protein antibody in a rat model of crush injury. Shock.

[18] Cuong NT (2013). Sivelestat improves outcome of crush injury by inhibiting high-mobility group box 1 in rats. Shock.

[19] He Q (2011). Crush syndrome and acute kidney injury in the Wenchuan earthquake. J. Trauma Inj. Infect. Crit. Care.

[20] Sever MS (2002). Clinical findings in the renal victims of a catastrophic disaster: The Marmara earthquake. Nephrol. Dial. Transplant..

[21] Basile DP, Anderson MD, Sutton TA (2012). Pathophysiology of acute kidney injury. Compr. Physiol..

[22] Sever MS, Vanholder R, Lameire N (2020). Acute kidney injury in active wars and other man-made disasters. Semin. Nephrol..

[23] Siddall E, Khatri M, Radhakrishnan J (2017). Capillary leak syndrome: Etiologies, pathophysiology, and management. Kidney Int..

[24] Coban YK (2012). Infection control in severely burned patients. World J. Crit. Care Med..

[25] Abid MR (2001). Vascular endothelial growth factor induces manganese-superoxide dismutase expression in endothelial cells by a Rac1-regulated NADPH oxidase-dependent mechanism. FASEB J..

[26] Petejova N, Martinek A (2014). Rhabdomyolysis (CC 2014). Crit. Care.

[27] Gillani S, Cao J, Suzuki T, Hak DJ (2012). The effect of ischemia reperfusion injury on skeletal muscle. Injury.

[28] Nathan C (2006). Neutrophils and immunity: Challenges and opportunities. Nat. Rev. Immunol..

[29] Honda M (2013). Intravital imaging of neutrophil recruitment in hepatic ischemia–reperfusion injury in mice. Transplantation.

[30] Nguyen HX, Lusis AJ, Tidball JG (2005). Null mutation of myeloperoxidase in mice prevents mechanical activation of neutrophil lysis of muscle cell membranes in vitro and in vivo. J. Physiol..

[31] Arecco N (2016). Elastase levels and activity are increased in dystrophic muscle and impair myoblast cell survival, proliferation and differentiation. Sci. Rep..

[32] Ziemkiewicz N, Hilliard G, Pullen NA, Garg K (2021). The role of innate and adaptive immune cells in skeletal muscle regeneration. Int. J. Mol. Sci..

[33] Chavakis T, May AE, Preissner KT, Kanse SM (1999). Molecular mechanisms of zinc-dependent leukocyte adhesion involving the urokinase receptor and β2-integrins. Blood.

[34] Taguchi K (2020). Carbon monoxide rescues the developmental lethality of experimental rat models of rhabdomyolysis-induced acute kidney injury. J. Pharmacol. Exp. Ther..

[35] Gois PHF (2016). Allopurinol attenuates rhabdomyolysis-associated acute kidney injury: Renal and muscular protection. Free Radic. Biol. Med..

[36] Tekşen Y, Kadıoğlu E, Koçak C, Koçak H (2019). Effect of hydrogen sulfide on kidney injury in rat model of crush syndrome. J. Surg. Res..

[37] Murata I (2013). Acute lethal crush-injured rats can be successfully rescued by a single injection of high-dose dexamethasone through a pathway involving PI3K-Akt-eNOS signaling. J. Trauma Acute Care Surg..

[38] Zhang BF (2017). Anti-high mobility group box-1 (HMGB1) antibody attenuates kidney damage following experimental crush injury and the possible role of the tumor necrosis factor-α and c-Jun N-terminal kinase pathway. J. Orthop. Surg. Res..

[39] Okubo K (2018). Macrophage extracellular trap formation promoted by platelet activation is a key mediator of rhabdomyolysis-induced acute kidney injury. Nat. Med..

[40] Murata I (2019). Icing treatment in rats with crush syndrome can improve survival through reduction of potassium concentration and mitochondrial function disorder effect. Exp. Ther. Med..

[41] Yang XY (2020). Ulinastatin ameliorates acute kidney injury induced by crush syndrome inflammation by modulating Th17/Treg cells. Int. Immunopharmacol..

[42] Haase H, Hebel S, Engelhardt G, Rink L (2006). Flow cytometric measurement of labile zinc in peripheral blood mononuclear cells. Anal. Biochem..

[43] Oley MH (2020). Hyperbaric oxygen therapy in managing systemic inflammatory response syndrome caused by ischemia–reperfusion injury following hand replantation and long-term outcomes: A report of two cases. Ann. Med. Surg..

[44] Saiwai H (2013). Ly6C+Ly6G− Myeloid-derived suppressor cells play a critical role in the resolution of acute inflammation and the subsequent tissue repair process after spinal cord injury. J. Neurochem..

